# Laparoscopic Gastric Resection for Gastric Cancer: Is Intracorporeal Anastomosis Necessary?

**DOI:** 10.12669/pjms.36.6.1915

**Published:** 2020

**Authors:** Ersen Ogun, Unal Ali Ekrem, Cemil Yuksel, Culcu Serdar, Salim Ilksen Basceken, Mercan Umit, Demirci Salim

**Affiliations:** 1 Ersen Ogun, Ankara University, General Surgery, Surgical Oncology, Ankara, Turkey; 2 Unal Ali Ekrem, Ankara University, General Surgery, Surgical Oncology, Ankara, Turkey; 3 Cemil Yuksel, Ankara University, General Surgery, Surgical Oncology, Ankara, Turkey; 4 Culcu Serdar Ankara Oncology Hospital, Surgical Oncology, Ankara, Turkey; 5 Bascseken Ilksen Salim Diyarbakır Oncology Hospital, Surgical Oncology, Diyarbakır, Turkey; 6 Mercan Umit, Ankara University, General Surgery, Surgical Oncology, Ankara, Turkey; 7 Demirci Salim, Ankara University, General Surgery, Surgical Oncology, Ankara, Turkey

**Keywords:** Stomach Neoplasms, Laparoscopy, Gastrectomy

## Abstract

**Background and Objective:**

In surgical dissection, laparoscopic approach and open techniques do not differ significantly, but there is still no consensus on how anastomosis should be performed in both cardia and distal gastric tumors. Anastomosis can be performed by laparoscopy-assisted mini-laparotomy or by intracorporeal suture techniques. In this study, we aim to present our four years of clinical experience and short-term surgical results from 133 cases in order to evaluate the necessity of laparoscopic anastomosis.

**Methods:**

This study was approved by Ethics Committee (No: 1-8-19, date: 14/01/2019). Patients who underwent curative resection with the diagnosis of gastric adenocarcinoma between January 2014 and January 2018 in the Ankara University Surgical Oncology Department were included in the study.

**Results:**

Of the 133 patients included in the study, 108 (81.2) were male and the mean age was 60.51 ± 12.0 years. The time of anastomosis was significantly longer in patients undergoing intracorporeal anastomosis (p = 0.021). The incidence of anastomotic leakage was significantly higher in the group undergoing intracorporeal anastomosis (p = 0.004).

**Conclusions:**

We think that esophagojejunostomy and jejunojejunostomy anastomoses in patients undergoing total gastrectomy should be performed with intracorporeal techniques in terms of benefit risk assessment. We believe that it is more feasible to continue the case with mini laparotomy when anastomosis is reached in patients who are planned to have gastrojejunostomy. In addition, in terms of intracorporeal anastomoses and advanced laparoscopic techniques, intracorporeal anastomoses performed in gastric cancer surgery for a laparoscopist who has completed the learning curve do not appear to be very different in terms of anastomosis safety.

## INTRODUCTION

The fourth most common malignancy in the world today is stomach cancer.^1^ The study of the biological characteristics of gastric cancer goes deeper, the chemical therapy for gastric cancer has made remarkable progress, but surgery is still the most important treatment for gastric cancer.^2^ Surgical resection with lymphadenectomy is the standard treatment for gastric cancer, but there is still controversy about the limits of lymphadenectomy.^3^-^6^ Apart from the extent of lymph node dissection, resections and forms of anastomosis in conventional gastric cancer surgery are now almost standardized. However, in laparoscopic cases, the situation is slightly different. The use of laparoscopy in gastric cancer was first as diagnostic laparoscopy to check operability and to determine if there was peritoneal disease.^7^ Subsequent laparoscopic assisted and partial laparoscopic cases followed by laparoscopic completed gastric cancer cases were reported.^8^-^10^ Recently, large series of laparoscopic gastrectomy procedures have been reported.^10^-^12^ However, the feasibility and safety of a completely laparoscopic gastrectomy has been controversial since it was first described.^12^-^14^

Nowadays it is an undisputed fact that gastrectomy can be safely performed laparoscopically in the competent hands, but the most important technical difficulties discussed are post-resection reconstruction. Especially in siewert-type II / III gastroesophageal junction tumors, surgical approach varies according to the clinics, technical difficulties arise during laparoscopic anastomosis stage for this type of adenocarcinoma. Especially esophagojejunostomy anastomosis is considered the most difficult technique in laparoscopic total gastrectonia due to the difficulty of manipulating stapler devices in a narrow space and the use of intracorporeal suture techniques above the level of hiatal aperture.^15^ In surgical dissection, laparoscopic approach and open techniques do not differ significantly, but there is still no consensus on how anastomosis should be performed in both cardia and distal gastric tumors. Anastomosis can be performed by laparoscopy-assisted mini-laparotomy or by intracorporeal suture techniques. Here we have two problems; whether intracorporeal techniques can be safely applied and the intra-postoperative returns. In this study, we aim to present our four years of clinical experience and short-term surgical results from 133 cases in order to evaluate the necessity of laparoscopic anastomosis.

## METHODS

Patients who underwent curative resection with the diagnosis of gastric adenocarcinoma between January 2014 and January 2018 in the Ankara University Surgical Oncology Department were included in the study. Patients who had local invasion and distant metastasis, multiorgan resection and patients whose clinicopathological data could not be reached were excluded from the study. Preoperative radiological staging and tissue diagnosis were decided for all patients. One hundred thirty three patients were included in the study. The patients were divided into two groups as laparoscopic and laparoscopic assisted cases. This study was approved by Ethics Committee (No: 1-8-19, date: 14/01/2019).

### Surgical Procedure

In our clinic, laparoscopic gastrectomy procedures due to gastric cancer have been performed with a standardized technique for six years and by an expert surgical team on gastric cancer. Before each operation, written informed consent was obtained from each patient about the procedures and scientific studies. None of the patients had bowel cleansing. All patients were taken to the operating table after an 8-hour fasting and prophylactic single dose antibiotherapy was performed (IV 1 gr cefazoline sodium). During the operation, the anesthesia team provided normothermia. Under general anesthesia, the patients were placed in the liloyd-davies position and the laparoscopy tower was positioned on the left shoulder. Just above the umblicus, a perpendicular incision with 10 vision and three working ports was entered from the locations in Fig.1. Standard lymph node dissection and resection were performed depending on the localization of the tumor without compromising the principles of open surgery. D1 dissection was performed according to the guidelines of the Japanese Gastric Cancer Society, which included 8a, 9, 10, 11 and 12a.^16^ In both total and subtotal groups, anastomosis was performed by laparotomy in one case and intracorporeal in one case.

### Laparoscopy Assisted Gastrectomy Procedure

In cases of subtotal gastrectomy, a mini laparotomy incision was used to remove the pathology specimen for anastomosis. The remnant gastric pouch was held with a clamp and pulled out of the abdomen. Afferent-efferent limb distinguish was performed laparoscopically before the jejunum was taken out of the abdomen. Jejunum was transected using linear stapler. Gastrojejunostomy anastomosis was performed by hand, double layer, with polyglactin and silk sutures along the large curvature without touching the stapler in the resection line. Esophagojejunostomy anastomosis was performed using circular stapler 25-27 through an 8-10 cm incision. Two support stitches were placed on the stapler line with silk suture. Jejunojejunostomy anastomosis was performed by hand anastomosis using side by side, double layer, outer layer silk and inner layer vicryl material. The Peterson pouch was individually sealed with silk sutures.

### Totally Laparoscopic Gastrectomy Procedure

Intracorporeal anastomosis technique, treitz ligament was explored and jejunum was found. The jejunum was transected by laparoscopic linear stapler and the mesentery was separated. In the subtotal gastrectomy group, laparoscopic linear stapler was performed antecolicly and gastrojejunostomy anastomosis was performed at the level of the large curvature of the stomach. Stapler entrances were closed in two layers using V-loc sutures. In total gastrectomy patients, laparoscopic linear stapler was performed side-by-side(overlap) esophagojejunostomy and the stapler entrances were closed with V-loc sutures. For jejunojejunostomy anastomosis, the small bowel loops were intercorporeally knotted with a single silk suture and secured together. Silk suture was taken from the umbilical trocar where the specimen was removed. Anastomosis was performed manually by using side by side, double layer, outer layer silk and inner layer vicryl material.

All anastomoses were Roux-en-Y, gastrojejunostomy anastomoses were routinely anticolic and esophagojejunostomy anastomoses were retrocolic. In all cases, esophageal anastomoses were tested with air water / methylene blue. The size of mini laparotomy was 5-6 cm in the subtotal gastrectomy group and 8-10 cm in total gastrectomy patients. The anterior abdominal wall fascia was closed using 1 or 0 double layer polydioxanone suture.

### Data Collection and Statistical Analysis

All these data were collected by our clinic’s experienced data collection assistant (general surgery and surgical oncology specialist). Gender, age, comorbidities, type of resection, duration of operation, length of hospital stay, complications associated with anastomosis (leakage, stenosis) were evaluated and noted from clinical files and electronic files in the hospital database. Numerical data are given as mean, plus-minus standard deviation. Student t test, man Whitney u test, x2 test or fisher exact test were used when appropriate for numerical and categorical data. P value less than 0.05 was considered significant. IBM SPSS version 23.0 was used for these statistical analyzes.

## RESULTS

Of the 133 patients included in the study, 108 (81.2) were male and the mean age was 60.51 ± 12.0 years. Male to female ratio was statistically significantly higher in patients undergoing extracorporeal anastomosis (p = 0.001>). There was no significant difference between the groups in terms of tumor localization, type of operation, disease prevalence and incidence of anastomosis stenosis. The time of anastomosis was significantly longer in patients undergoing intracorporeal anastomosis (p = 0.021). The incidence of anastomotic leakage was significantly higher in the group undergoing intracorporeal anastomosis (p = 0.004). Clinicopathological and postoperative results in the groups of intra and extracorporeal anastomosis have summarized in Table-I.

**Table-I:** Comparison of Clinicopathological and postoperative results between the groups of intra and extracorporeal anastomosis.

Variables	Total (n=133)	Intracorporeal anastomosis (n=42)	Extracorporeal anastomosis (n=91)	p value
Age	60.51±12.0	60.24±14.48	60.64±10.87	0.860
Gender(male)	108(81.2)	25(59.5)	83(91.2)	0.001>*
***Tumor localisation***				
Distal	54(40.6)	17(40.5)	37(40.7)	0.479
Central	23(17.3)	5(11.9)	18(19.8)	
Proximal	56(42.1)	20(47.6)	36(39.6)	
***Operation***				
SG	60(45.1)	18(42.9)	42(46.2)	0.434
TG	73(54.9)	24(57.1)	49(53.8)	
***TNM Stage***				
Early Stage	50(37.6)	17(40.5)	33(36.3)	0.390
Locally Advance	83(62.4)	25(59.5)	58(63.7)	
Time of anastomosis(min)	40.52±6.75	42.50±4.84	39.61±7.31	0.021*
Anastomotic leakage	11(8.3)	8(19)	3(3.3)	0.004*
Anastomotic Stenosis	6(4.5)	3(7.1)	3(3.3)	0.282

Numerical values are given as mean ± standard error. SG: Subtotal Gastrectomy, TG: Total Gastrectomy.

In the subgroup of patients undergoing subtotal gastrectomy, no significant difference was found on the time of anastomosis and the incidence of anastomotic leakage between intracorporeal and ekstracorporeal anastomosis. The incidence of anastomotic stenosis was high, although not statistically significant, in patients undergoing intracorporeal anastomosis (p= 0.070). Times of anastomosis and postoperative and long-term complications of intra corporeal versus extracorporeal anastomosis in subtotal gastrectomy subgroup has been summarized in Table-II.

**Table-II:** Comparison of times of anastomosis and postoperative and long-term complications of intracorporeal versus extracorporeal anastomosis in subtotal gastrectomy subgroup.

Variables	Total (n=60)	Intracorporeal anastomosis (n=18)	Extracorporeal anastomosis (n=42)	p value
Time of Anastomosis (min)	40.12±7.44	41.87±4.72	39.37±8.28	0.236
Anastomotic leakage	3(5)	2(11.1)	1(2.4)	0.212
Anastomotic Stenosis	4(6,7)	3(16.7)	1(2.4)	0.070

Numerical values are given as mean ± standard error.

In the subgroup of patients undergoing total gastrectomy, no significant difference was found on the incidence of anastomotic stenosis between intracorporeal and extracorporeal anastomosis. The time of anastomosis and the incidence of anastomotic leakage were significantly higher in the patients undergoing intracorporeal anastomosis (p= 0,039; p= 0,012). Times of anastomosis and postoperative and long-term complications of intra corporeal versus extracorporeal anastomosis in total gastrectomy subgroup is summarized in Table-III.

**Table-III:** Comparison of times of anastomosis and postoperative and long-term complications of intracorporeal versus extracorporeal anastomosis in total gastrectomy subgroup.

Variables	Total (n=73)	Intracorporeal anastomosis (n=24)	Extracorporeal anastomosis (n=49)	p value
Time of Anastomosis (min)	40.85±6.15	42.96±4.98	39.82±6.45	0.039*
Anastomotic Leakage	8(11)	6(25)	2(4.1)	0.012*
Anastomotic Stenosis	2(2.7)	0(0)	2(4.1)	0.447

Numerical values are given as mean ± standard error.

## DISCUSSION

Intracorporeal suture techniques are classified as advanced laparoscopic techniques that require more experience. Although series have been reported in the literature, complication rates associated with anastomosis are similar to those of open techniques, although this may not be seen in practice. Conventional anastomosis techniques are more reliable than laparoscopic intracorporeal techniques since they are familiar and experienced throughout the life of surgeons.

### Comparison of subtotal gastrectomy patients

In our study, there was no difference in the time to perform gastrojejunostomy and jejunojejunostomy from a 5 cm incision which is mandatory for removal of pathology specimen in subtotal gastrectomy patients. The fact that open and laparoscopic anastomoses were not different in terms of duration was associated with the ability of subtotal gastrectomy anastomoses to be easily performed laparoscopically if the remaining gastric pouch was large enough. It should be noted that none of the patients need to expand laparotomy incisions for anastomosis. In one patient, gastric anastomosis was performed on the afferent limb side due to confusion, and intraoperative reanastomosis was performed when seen during the control. Especially in patients with long anterior or posterior diameter of the thorax or obese, confusion may occur as to which of the afferent loops during mini-laparotomy incision. This complication was caused by the mixing of the afferent and efferent parts during the manipulation of the jejunum pulled from the small incision. In order to prevent this situation, we believe that the laparoscopic transexion of the jejunum and the fixation of the efferent loop into the stomach with intracorporeal suture may be useful in the latest cases of our series.

In a study of 100 cases comparing totally laparoscopic and laparoscopic assisted distal gastrectomies, anastomotic leakage rates were found to be 2% in both groups.^17^ When the cases in our study were evaluated in terms of anastomosis-related complications, it was found that there was no statistically significant difference in anastomosis leakage in gastrojejunostomy anastomoses (p = 0.212). In terms of stenosis, our study revealed that stenosis was significantly higher in laparoscopic anastomoses (p = 0.070). Based on these results, if a laparotomy is planned to remove the specimen in subtotal / distal gastrectomy, anastomoses with open technique do not have any disadvantage for the patient. Although there are no extensive series of studies in the literature, we think that intracorporeal anastomosis can provide significant benefit in patients with subtotal gastrectomy performed by natural orifice specimen extraction (NOSE) method using transrectal and transvaginal tract.^18^,^19^

### Comparison of total gastrectomy patients

In patients undergoing total gastrectomy, open anastomosis technique, esophageal purse-string suturing is only possible through an 8-10 cm incision and is technically difficult especially in patients whose abdominal esophagus is short or completely displaced into diaphragmatic cruses. However, in patients undergoing total gastrectomy, a 10 cm incision is performed only for anastomosis, which results in the loss of laparoscopy gains for the patient. The fact that total gastrectomy and D2 lymph node dissection can be performed through an approximately 15 cm incision extending from the xiphoid to the umbilicus in open surgery shows that the choice of such a large incision for laparoscopic cases is inconvenient. In order to perform esophagojejunal anastomosis laparoscopically, techniques such as using transorally inserted anvil (OrVil™), overlap technique and reverse anvil technique have been described.^20^-^22^ In case series using transorally inserted anvil, a wide leak percentage range of 0.5-16.7% has been reported by Zuiki et al. In the series of 52 cases reported, this rate was reported as 1.9% and the rate of anastomosis stricture was as high as 21%.^23^ On the other hand, overlap anastomosis was performed predominantly. In another series, the rate of anastomosis leakage was determined as 4.8%.^24^ In a study comparing 124 cases selected from 1258 laparoscopic gastrectomy patients and 3268 open gastrectomy patients using all three of OrVil™, overlap and anvil techniques, anastomosis leakage rate was 2.4% and anastomosis stricture rate was 1.6%.^25^ In our cases, we used the esophagojejunostomy technique performed side by side with linear stapler described by Inaba et al.^26^ Anastomotic leakage and stenosis caused by this technique was 2.7% in accordance with the literature. In terms of case duration, totally laparoscopic total gastrectomy cases were presented in two separate studies and the mean operation time was 140-230 minutes.^25^,^26^ In our study, total laparoscopic total gastrectomy cases had a mean operation time of 159 minutes and were similar to those of the literature. When we look at the results of laparoscopic subtotal gastrectomy, the rate of anastomosis leakage was reported as 5% in a study in which completely laparoscopic cases were presented. However, the low number of cases in this study (n = 20) and the lack of clarity as to whether intra-abdominal abscess was associated with anastomotic leakage in two patients may indicate that the results of the study are far from guiding. In our study, anastomosis leakage was found to be significantly higher in esophagojejunostomy anastomoses (p = 0.012). Especially in cases of cardia tumors, when esophageal resection is performed liberally in order to provide a clean surgical margin, intraabdominal esophagus has to be completely removed and the esophagus is displaced from the hiatal gap to the posterior-thoracic. Especially in these patients, there are serious difficulties when closing the patency of the overlap anastomosis with linear stapler. This explains the excess of anastomotic leakage. When the stenosis rates were examined, no difference was found between laparoscopic cases with open anastomosis technique using circular stapler. The duration of anastomosis was significantly higher in intracorporeal anastomosis (p = 0.039).

### Limitations of our study

We do not have a balanced group distribution and the study is retrospective. A prospective, randomized and controlled study can be planned to have more objective results. In addition, if the number of patients who underwent intracorporeal anastomosis is increased, statistically more meaningful and reliable results will be obtained.

In the light of this information, we think that esophagojejunostomy and jejunojejunostomy anastomoses in patients undergoing total gastrectomy should be performed with intracorporeal techniques in terms of benefit and risk assessment. In patients who underwent subtotal and distal gastrectomy, it is seen in our study that the incision to remove the pathology specimen allows anastomosis to be performed easily. We believe that it is more feasible to continue the case with mini laparotomy when anastomosis is reached in patients who are planned to have gastrojejunostomy. In addition, in terms of intracorporeal anastomoses and advanced laparoscopic techniques, intracorporeal anastomoses performed in gastric cancer surgery for a laparoscopist who has completed the learning curve do not appear to be very different in terms of anastomosis safety.

### Authors’ Contribution:

**OE:** Designed study, did manuscript writing and did final approval of manuscript, made surgical operations, responsible and accountable for the accuracy or integrity of the work.

**UAE:** Final check of the article.

**CY:** Manuscript editing and proofreading.

**CS:** Data collection

**SIB:** Manuscript editing and proofreading.

**MU:** Statistical analysis.

**DS:** Made surgical operations.
